# Elucidation of molecular and functional heterogeneity through differential expression network analyses of discrete tumor subsets

**DOI:** 10.1038/srep25261

**Published:** 2016-05-03

**Authors:** Rutika R. Naik, Nilesh L. Gardi, Sharmila A. Bapat

**Affiliations:** 1 National Centre for Cell Science, NCCS Complex, Pune 411007, INDIA

## Abstract

Intratumor heterogeneity presents a major hurdle in cancer therapy. Most current research studies consider tumors as single entities and overlook molecular diversity between heterogeneous state(s) of different cells assumed to be homogenous. The present approach was designed for fluorescence-activated cell sorting-based resolution of heterogeneity arising from cancer stem cell (CSC) hierarchies and genetic instability in ovarian tumors, followed by microarray-based expression profiling of sorted fractions. Through weighted gene correlation network analyses, we could assign enriched modules of co-regulated genes to each fraction. Such gene modules often correlate with biological functions; one such specific association was the enrichment of CD53 expression in CSCs, functional validation indicated CD53 to be a tumor-initiating cell- rather than quiescent CSC-marker. Another association defined a state of poise for stress-induced metastases in aneuploid cells. Our results thus emphasize the need for studying cell-specific functionalities relevant to regeneration, drug resistance and disease progression in discrete tumor cell fractions.

Elucidating the molecular mechanisms of cell behavior and functions is a major goal in biology. However even today, quantification of averaged measures from pooled cell populations in a sample is preferred over targeted studies with isolated, similar cell groups or single cells^1^. This fails to capture outlier behavior that could explain subtle cell functions such as different cell fates, transitions from normal to diseased states, drug resistance, etc. Intratumor heterogeneity attributed to non-genetic, tissue-specific regulatory mechanisms as well as genetic variations that give rise to phenotypic, molecular and functional diversity, is considered as a confounding factor in such studies and in therapy^2^. A dominant non-genetic variance is realized from the cancer stem cell (CSC) hypothesis that posits tumor generation and establishment from a single transformed ‘stem-like’ cell/clone^3^. Classical stem cell hierarchies and their variations in cancer are realized to optimize cell resources towards cell survival and long-term tissue homeostasis by producing different cells at various levels of cell fate commitment and functionality. Several of the current CSC markers including CD133, CD44, CD24, CD117, CD34, CD38 are derived from earlier studies establishing their expression in normal tissue stem cells^4^,^5^,^6^,^7^. In ovarian cancer, despite reports of diverse CSC markers including CD44 and CD117^8^, MYD88^9^, ABCG2^10^, CD34^11^, CD24, CD90, CD133^12^,^13^, a contradiction for their exclusive association is raised through residual regenerative potential in non-CSC tumor fractions^14^. In their quest for CSC identification, most reports also ignore the fact that each level of the regenerative hierarchy is a critical determinant of tumor identity. In an earlier report we achieved resolution of the tumor regenerative hierarchy by determining the quenching dynamics of a vital membrane label PKH26/67 through flow cytometry as cells retaining either, (i) high intensity of the fluorophore (PKH^hi^), (ii) partial intensity (PKH^lo^) or (iii) undergoing complete quenching (PKH^neg^^15^). Functional, regenerative potential of label-chase demarcated fractions was also extensively examined through *in vitro* and *in vivo* asays that established PKH^hi^ cells to be CSCs, PKH^lo^ as progenitors and PKH^neg^ cells to constitute the differentiated tumor bulk respectively^15^,^16^. Importantly, these studies also assigned a definitive role to genetic instability (aneuploidy) in drug resistance and long-term tumor survival.

The introduction of molecular heterogeneity through genetic instability further fosters long-term survival of tumor cells through clonal evolution and selection by sequestration of genetic diversity for tumor adaptation that often culminates in therapeutic failure^17^. Aneuploidy leads to selective adaptive changes ensuring tumor survival under stress^18^. The genetic variance imposed by aneuploidy is also recognized to be a major player in tumor dormancy, yet has been a challenge to elucidate^19^,^20^,^21^. In the present study, towards further molecular understanding of the dynamics of tumor heterogeneity, we resolved and characterized discrete cellular fractions based on the regenerative hierarchy and genetic instability by combining flow sorting with gene expression microarrays in a xenograft model of ovarian cancer. Further analyses and functional validation generated knowledge relating to regeneration associated markers and molecular pathways of drug resistance and residual disease that could contribute to improvement of present day therapy.

## Methods

### Cells culture, xenograft generation, flow cytometry and sorting

A4 cells used in the study were established from malignant ascites of a patient diagnosed with grade 4 serous ovarian adenocarcinoma and cultured in MEM medium with 5% FBS and 1% NEA^22^. 2.5 × 10^6^A4 cells were stained with 2 μM PKH67 (PKH67-GL; Sigma-Aldrich) for 7 minutes, washed with ice-cold MEM(E) medium, and injected subcutaneously in NOD/SCID mice. Mice were bred and maintained at the NCCS Experimental Animal Facility. All experimental procedures and protocols performed in accordance with the laws and policies and approved by the NCCS Institutional Animal Ethics Committee. Tumors were harvested after 4 weeks and processed for flow cytometry analysis and sorting. Tumor fractions were sorted at 99% purity based on label intensity as PKH^hi^, PKH^lo^, PKH^neg^. Fractions were gated considering freshly labeled and unlabeled tumor cells were used as positive and negative controls respectively^15^. Additionally, tumor cells were stained with Hoechst (45 mins) and Pyronin Y (45 mins) at 37 °C before sorting cell fractions based on DNA-RNA content and cell cycle phases, where fractions were defined as euploid (G0 + G1) and aneuploid (G0 + SG2M) since segregation of entire fraction required intracellular staining of cyclin B1. Paclitaxel was administered intraperitoneally 25 mg/kg body weight of mice in two regimes as 3 doses (3D) at an interval of 48 hrs followed by 1 recovery cycle) or 6D (2 cycles of 3D)^16^. Seven aneuploid clones from paclitaxel-treated A4 xenografts (5 doses of 25 mg/kg body weight mice at an interval of 7 days) were established following cell sorting and culture. Cell sorting, data acquisition and analysis were achieved using BD FACS Aria II Sorp (BD Biosciences) and FACS Diva software version 6.0.

### Microarray data analysis

Microarray hybridization (Agilent Human Whole Genome 8 × 60 K Array) and data acquisition were carried out using standard procedures. Briefly, samples were labeled using the Agilent Low Input RNA amplification kit and hybridized to Agilent Human Whole Genome 8 × 60 k Array using Agilent *In situ* Hybridization kit. Triplicate datasets of each sorted fraction (PKH^hi^, PKH^lo^, PKH^neg^, euploid and aneuploid) were analyzed using Agilent Genomic Work Bench. Each data were normalized using median over entire array normalization method in BRB array tools v3.8.0^23^. Genes were filtered by class comparison method using criteria of p-value > 0.05 of univariate test, minimum fold change in which 20% of the expression data values have at least 1.5 fold change. Class comparison derived differential genes were subjected to two-way hierarchical clustering using MeV_4_8_1 software. Class comparison derived 5216 differential genes across 5 tumor sorted fractions (PKH^hi^, PKH^lo^, PKH^neg^, euploid and aneuploid) each in triplicate i.e 15 sample dataset were subjected to Weighted Gene Correlation Network Analysis (WGCNA) for derivation of functionally correlated genes. Pearson correlation coefficient was calculated between all pairs of genes across 5 tumor sorted microarray samples. The correlation matrix generated was raised to power 6, producing weighted correlation matrix. Weighted correlation matrix converted into topological overlap (TO), which not only measure the correlation between two genes but also extent of overlap across the weighted network. Genes were clustered based on TO values and dendrogram were plotted using Dynamic tree cut algorithm. Representative expression profile (module eigengene values) for each module was generated based on first principle component of genes containing thatparticular module. The resulting MEs were used to identify representative signature genes for each module, based on correlation criteria. Unsupervised Principle Component Analyses (PCA) was carried out for resolution of overlap between tumor fractions across 3 samples of five tumor fractions^24^,^25^. AracNe (Accurate Cellular Networks) was used to predict the interaction network the MI was fixed at 0.20; data processing inequality was set at 0.10, networks were visualized using Enrichment map in Cytoscape_v2.8.2^26^. Pathway analysis was done using DAVID Bioinformatics Resource v6.7^27^.

### RNA isolation, cDNA synthesis and qPCR quantification

Sorted cells were subjected to standard trizol- based RNA isolation and cDNA synthesis protocols. qPCR analyses with specific gene primers were carried out with Step one plus in 96- well plate format using SYBR Green Mix (Life Technologies). Changes in threshold cycle (CT) values were calculated as: ΔCT = CT (test) − CT (control); fold difference was calculated as: fold difference = 2−Δ(ΔCT). GAPDH expression was used for normalization; non-template controls accounted for possible contaminating DNA in reaction mixtures. Primer sequences are available on request.

### Functional assays for assessment of proliferative, regenerative and migratory potential

#### Clonogenecity

Tumor sorted fractions were plated in 96 well plate with cells (500 cells/well). Assays were terminated on Day 14 and plates subjected to crystal violet staining. Cells were washed with PBS, fixed with chilled 3% paraformaldehyde and incubated with 0.05% crystal violet dye for 2 hours. Images were captured by camera (Olympus). Clonogenic potential was counted by using Image J software. For drug treated fractions relative colony counts were established after normalizing with those from corresponding A4 control tumor fractions.

#### Soft agar assay

*In vitro* tumorigenecity and anchorage independent growth was assayed using standard methods. For colony formation, tumor fractions were sorted, suspended in 0.5% agarose at a concentration of 5000 cells and seeded in 35 mm plates containing a basal layer of 1% agarose. Colonies were counted on Day 14 under Olympus IX71 microscope at 10X magnification.

#### Spheroid formation assay

Each tumor sorted fractions was seeded in ultra low attachment plate at a density of 5 × 104 cells/well and cultured in MEM containing 1% serum. Spheroids were counted on Day 14 and images were captured on Olympus IX71 microscope.

#### Scratch assay

Tumor sorted fractions were plated in 96 well plate at a density of 1000 cells/well, media was changed every 48 h and cells grown till confluency. Wound was inflicted with a pipette tip, washed twice with 1XPBS to remove floating cells and serum-free MEM medium was added. Wound healing was monitored for 72 hours; Images were captured on Olympus IX71 microscope and analyzed by TScratch Software.

#### Matrigel Migration assay

5000 sorted cells were seeded in a matrigel chamber (Bd Biosciences) in 24 well plate. Media containing serum (MEM + 5% FBS) was used as chemoattractant in the lower well. Cells migrating and attaching to the bottom of the plate were stained with crystal violet and quantified after 48 hours.

#### Limiting dilution assay

Tumor-initiating potential was evaluated by injecting tumor cells (5000, 10000 or 20000 cells; 1:1 - matrigel:1XPBS) subcutaneously in NOD/SCID mice. Tumor formation was monitored for up to 1 month after injections. Stem cell frequency/tumor-initiating potential was calculated using the ELDA software^28^.

### Immunoflorescence (IF) and Immunohistochemical (IHC) staining

2 × 10^3^ sorted euploid and aneuploid cells from A4 xenograft tumors were seeded on cover-slips and subjected to scratch assay as described earlier^24^. After 48 hours cells were fixed with 4% paraformaldehyde, blocked with 10% goat serum and subjected to IF staining. Thus, cells were incubated with primary antibody against Moesin (1:100; Abcam) for 1 hour followed by corresponding secondary antibody, and subsequently with Falloidin. Images were acquired and analyzed on confocal microscope (Leica, Germany). Standard protocols were used for IHC as described earlier^24^. Briefly, 5 μ thick sections of A4 xenografts embedded in paraffin were deparaffinized and hydrated using an alcohol - water gradient. Subsequently sections were washed twice in 1XPBS, blocked with 10% goat serum for 30 min and incubated with primary Moesin/Slug antibody (1:100 dilution) overnight. Internal peroxidase activity was neutralized using 0.3% H_2_O_2_ followed by incubation with horseradish peroxidase (HRP) conjugated secondary antibody for 30 minutes. Color was developed using DAB (3,3′-diaminobenzidine) and sections counterstained with Hematoxylin. Finally, slides were dehydrated, xylene cleared, mounted and imaged using CellD software for Olympus IX71. Analysis and quantification of frequency of cells associated with protein expression was performed with ImageJ software.

### Statistical analysis

Unless mentioned otherwise, all experiments were carried out in triplicate; data is expressed as mean ± SEM of at least three independent experiments. The significance of difference in the mean values was determined using two-tailed Student’s t test *p < 0.05; **p < 0.01; ***p < 0.001.

## Results

### Genes-modules-pathway correlations with tumor fractions

**Figure 1:**
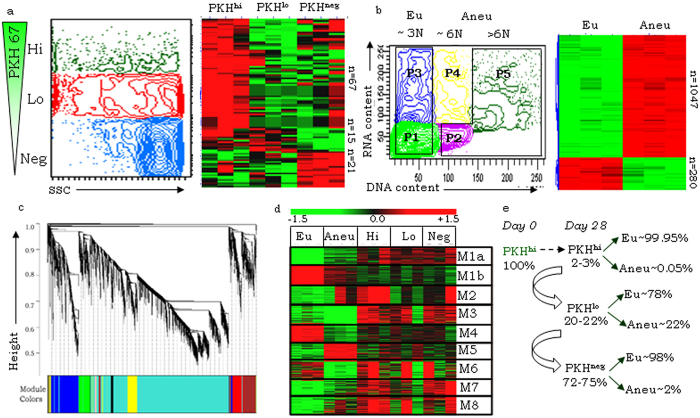
Identification of differential or enriched genes and modules across heterogeneous tumor fractions. (**a,b**) left panels - Representative FACs profiles for PKH derived cancer stem cell (CSC) hierarchy and Hoechst PyroninY-based DNA content analysis respectively, where P1- EuG0, P2-AneuG0, P3-EuG1, P4-(EuSG2M + AneuG1), P5-AneuSG2M, denoting sorted euploid (P1 + P3) and aneuploid (P2 + P5) fractions; (**a,b**) -right panels - Representative heat maps of differential genes obtained through class comparison across various PKH- and ploidy- based fractions respectively, red and green colors correspond to up-regulated and down-regulated genes; (**c**) WGCNA-based cluster dendrogram identifies modules of correlated genes in the differential expression data; (**d**) Heatmap of top 50 highly correlated genes from each WGCNA module; (**e**) Flow-chart depicting frequency of fraction turnover in xenografts from Day 0 to Day 28 based on our earlier studies^15^,^16^.

The regenerative hierarchy in xenografts demarcated on a basis of quenching kinetics of the membrane fluorophore PKH, identified three fractions as described above. Briefly, the PKH^hi^ fraction represents CSCs, PKH^lo^ includes tumor progenitors and PKH^neg^ cells represent differentiated component of the tumor. Alternatively, variability in the cellular DNA content identified two discrete tumor fractions representing euploid and aneuploid cells (Fig. 1a,b-left panels). Class comparison of gene expression datasets from each of these fractions indicated association of definitive expression patterns (Fig. 1a,b-right panels). 5216 differentially expressed genes were identified across the five cell fractions that could be clustered in 8 modules using Weighted Gene Correlation Network Analysis (WGCNA; Fig. 1c); each module represents a set of strongly correlating genes that do not correlate with the genes in other modules (Supplementary Table 1).

Unsupervised, Principal Component Analysis of the top, most significant 50 WGCNA module genes (n = 439) distinctly segregated euploid from other data; aneuploid datasets exhibited low distance similarity with two PKH^lo^ datasets, while data of the PKH^hi^ fractions clustered closely with that of PKH^neg^ (Supplementary Fig. 1a). Very importantly, the WGCNA modules were also observed to be differentially enriched across various cell fractions (Fig. 1d; Supplementary Fig. 1b) suggesting an association of distinct molecular expression profile with each fraction. With a view to identify such differentially expressed gene signatures across distinct cellular subsets, we first eliminated the most unlikely associations based on our earlier studies^15^,^16^ (Fig. 1e) that suggest an overlapping, heterogeneous nature of the PKH and DNA content based subsets. The most important and relevant considerations thus identified include–

- (i) PKH^hi^ and PKH^neg^ are almost entirely euploid and constitute non-cycling states, and
- (ii) dormant aneuploid cells are confined to the PKH^lo^ fraction (Fig. 1e).

Thus associations predicted from our analyses that seemed unlikely included enrichment of modules M3, M7, M8 in PKH^hi^ and PKH^neg^ but not in euploid data; M1b and M4 in euploid fractions but not in PKH^hi^ or PKH^neg^ data. On the other hand, the most likely associations predicted included enrichment of module M2 expression in the PKH^hi^ fraction, and an anti-correlative association of M5 and M6 module genes in euploid and aneuploid cells respectively.

**Figure 2:**
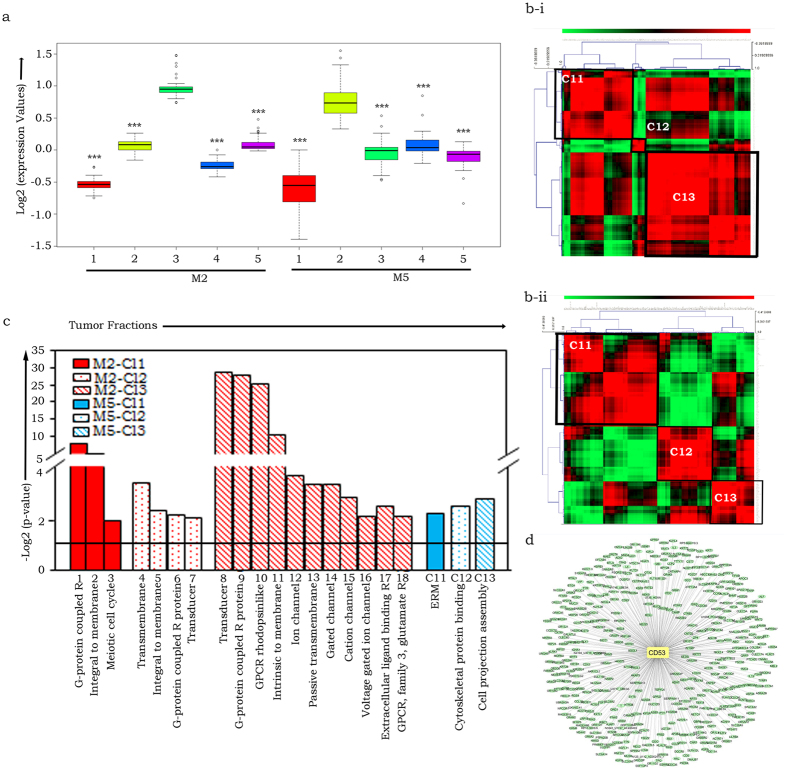
Association of expression of M2 and M5 modules with PKH^hi^ and aneuploid tumor cell fractions respectively and prediction of functional identities. (**a**) Box-plot representation of Log2 expression (intensities) of M2 and M5 genes across tumor fractions, where 1- Euploid, 2- Aneuploid, 3- PKH^hi^, 4- PKH^lo^, 5- PKH^neg^, p-values established using Welch two sample t-test, ***p < 2.2e-16; (**b**) Pearson correlation matrix plots that identify three distinct gene clusters *viz.* Cl1 (Cluster 1), Cl2 (Cluster 2) and Cl3 (Cluster 3) each in modules M2 (**b-i**) and M5 (**b-ii**) of PKH^hi^ and aneuploid datasets respectively; (**c**) DAVID pathway analysis of correlating gene clusters, Y-axis represents pathway significance for module M2 (represented in red colour patterns) in which Cl1 (1-G-protein coupled receptor, 2-Integral to membrane signaling, 3-Meiotic cell cycle), Cl2 (4- Transmembrane signaling, 5- Integral to membrane signaling, 6- G-protein coupled receptor protein signaling pathway, 7- Transducer signaling pathway), Cl3 (8- Transducer signaling, 9- G-Protein coupled receptor protein signaling pathway, 10- GPCR, rhodopsinlike, 11- Intrinsic to membrane, 12- Ion channel activity, 13- passive transmebrane transporter activity, 14- Gated channel activity, 15- Cation channel activity, 16- Voltage-gated ion channel activity, 17- Extracellular ligand-binding receptor, 18- GPCR, family 3, glutamate receptor) and module M5 (represented in blue colour patterns) in which Cl1 (Ezrin/radixin/moesin ERM), Cl2 (cytoskeletal protein binding), Cl3 (Cell projection assembly); (**d**) AracNe based gene interaction network across M2 genes with CD53 as node.

To further establish unequivocal associations, gene expression intensities of M2 and M5 module genes were compared across tumor fractions. Distinct up-regulation affirmed association of M2 and M5 genes with PKH^hi^ and aneuploid fractions respectively (Fig. 2a). Pearson analysis to identify highly correlating genes within modules M2 and M5 further revealed 3 distinct clusters in each module, that suggest an association with specific functionalities (Fig. 2b,c; Supplementary Table 2). Thus, within the PKH^hi^ datasets, DAVID pathway analyses^27^ assigned the strong correlation between M2 associated gene clusters Cl1 & Cl3 due to shared functions of G-protein coupled signaling, although Cl1 is additionally involved in meiotic cell cycle and Cl3 in transporter activity pathways. Strikingly, the smaller Cl2 cluster negatively correlates with Cl1 & Cl3, predicatively to maintain homeostasis of G-protein signaling. Of the three module M5 clusters in aneuploid datasets, clusters Cl2 and Cl3 positively correlated with each other due to shared functionalities associated with cytoskeletal proteins and cell projection assembly components in cell division, and negatively correlated with Cl1 (cytoskeletal remodelling by ERM proteins). Intriguingly, this suggests a decoupling of cell division from cell migration.

### Module M2 analysis and validation assigns CD53 functions

**Figure 3:**
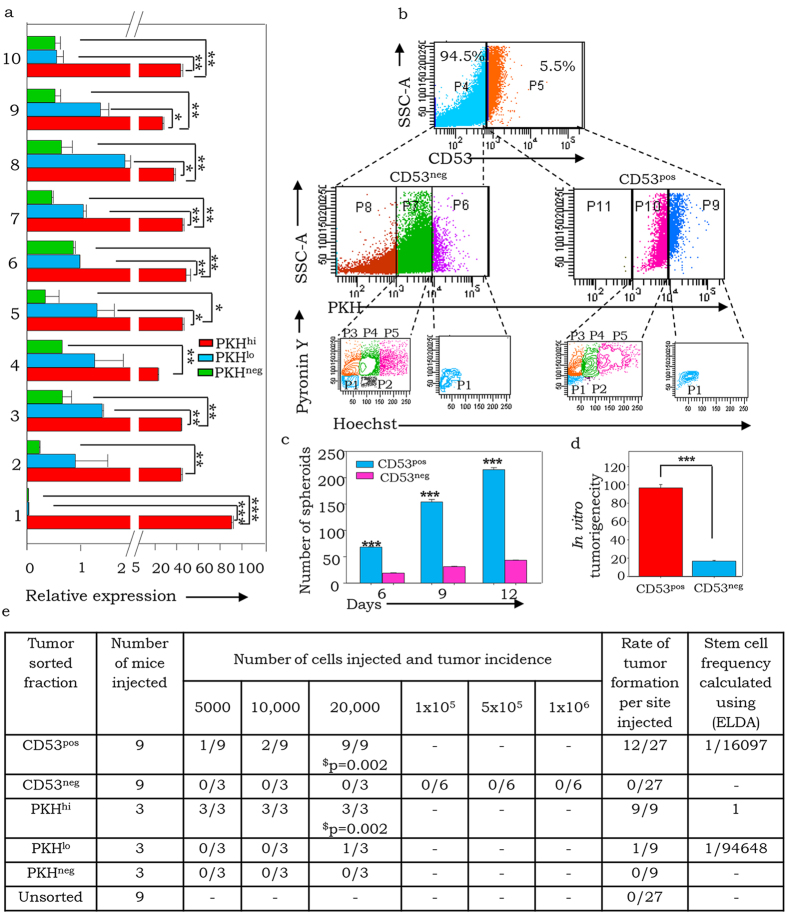
Module 2 genes exhibit stem cell functionalities and expression of CD53 is associated with regenerative potential within tumor cells. (**a**) Real time PCR quantification of M2 genes (1- CD53, 2-CD84, 3- KRT1, 4- CCL18, 5-CXCL6, 6- MMP26, 7- S1007A, 8- SKT3B, 9- PRPY1, 10- TRAT1) in A4 tumor sorted PKH^hi^, PKH^lo^, PKH^neg^ cells; (**b**) Representative FACS profile of CD53 expression in A4 xenograft tumor (upper panel) and across PKH based CSC hierarchy (middle panel) and Hoechst Pyronin Y based cell cycle profiling of PKH^hi^ and PKH^lo^ fraction across CD53 expression where fraction P1-P5 are same as mentioned in Fig. 1a; (**c**) Graphical representation of functional potential of sorted populations (CD53^neg^; CD53^pos^,) in assays for: spheroid formation; (**d**) - anchorage-independent clonogenecity; (**e**) Limiting dilution assay for *in vivo* tumorigenic potential of tumor sorted CD53^neg^; CD53^pos^, PKH^hi^, PKH^lo^ and PKH^neg^ fractions, where Stem cell frequency was estimated based on number of regenerated tumors in limiting dilution assay using ELDA software^28^, $-p-values based on Students t-test between CD53^pos^vs CD53^neg^ and PKH^hi^ vs PKH^neg^.

Literature-based functional annotation assigned further relevance to expression of M2 genes in the PKH^hi^/CSC fraction through their known involvement in stem cell functioning and differentiation, cell survival, cell adhesion and migration (Supplementary Table 3). Network analyses of these genes revealed the cell surface tetraspanin molecule CD53 to be a major hub with several interacting partners (Fig. 2d). In validating expression of some of the M2 genes, CD53 exhibited a striking exclusive association with the PKH^hi^ fraction, although the protein was associated not only with PKH^hi^ but also with euploid and G2M-growth arrested aneuploid PKH^lo^ fractions (Fig. 3a,b; Supplementary Fig. 2a–c). CD53 was also expressed in xenografts derived from other serous ovarian cancer cell lines including CAOV3, OVMZ6, OVCAR3 and OV90 that suggests a pan-ovarian cancer association (Supplementary Fig. 2d).

In further probing for functional correlates of this expression, we observed differences in regenerative potential of CD53 expressing (CD53^pos^) vs. CD53 lacking (CD53^neg^) cells when assayed for spheroid formation, *in vitro* tumorigenecity and *in vivo* limiting dilution assays for tumorigenecity potential (Fig. 3c–e). Similar evaluation of PKH derived fractions in the latter assay indicated PKH^hi^ cells to exhibit maximal regenerative ability [estimated as 1 in ELDA -Extreme Limiting Diluting Assay software^26^ that is useful in comparing stem cell frequencies], while PKH^lo^ cells have a much lower potential (1/94648), and PKH^neg^ cells are devoid of regenerative abilities. Since CD53 expression is associated with only the PKH^hi^ and PKH^lo^ cell fractions of tumors (Fig. 3b, middle panel) and exhibits an intermediate stem cell frequency (1/16097), this strongly suggests an association with regenerative functions within a tumor. Thereby, these results suggest CD53 to be a putative marker for tumor initiating/regenerative potential.

### Aneuploid cells harbor latent metastatic potential

**Figure 4:**
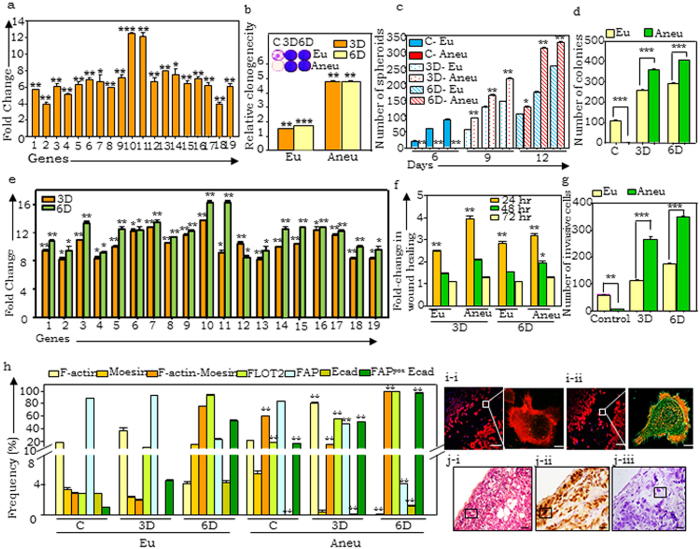
Aneuploid cells exhibit active cytoskeletal remodeling which is enhanced on paclitaxel exposure. (**a**) Real time PCR quantification of expression of M5 genes (1- DST, 2- VAMP3, 3- PKM2, 4- F2RL2, 5- ASPH, 6- MCM4, 7- PLEC1, 8- SNRPB, 9- ARHGDIA, 10- MSN, 11- FLOT2, 12- PRICKLE1, 13- RDH10, 14- ABL2, 15- WWTR1, 16- BRCC2, 17-MYO1E, 18-PSCD3, 19-F3) in aneuploid fraction of A4 naïve (untreated) tumor, fold-change gene expression is calculated with respect to that in the euploid fraction; (**b**) Anchorage dependent clonogenecity of tumor sorted fractions - inset shows crystal violet stained images, (**c**) Quantification of spheroid formation potential of sorted euploid and aneuploid cells (Eu and Aneu; blue and red color patterns respectively) in untreated tumors (C) or exposed to either 3 or 6 doses of paclitaxel (3D and 6D respectively); (**d**) *In vitro* tumorigenecity or similarly sorted populations; (**e**) Real time PCR quantification of expression of M5 genes in similarly sorted populations where fold-change expression in aneuploid cells is estimated with respect to that in the euploid fractions for each gene (1-19 as mentioned in (a), (**f**) Fold-change wound healing/migratory efficiency cells; (**g**) Quantification of matrigel invasion assay; (**h**) Graphical representation of flow cytometry analysis of Moesin, F- actin, flotillin, FAP and E-cadherin expression in untreated and paclitaxel treated tumors; (**i**) Representative immunostaining images of scratch assays in (**i**-i) euploid and (**i**-ii) aneuploid fractions (scale bar- 10 μ)- insets represent migratory cells (red and green denotes F-actin and Moesin respectively, scale bar-100 μ); (**j**) Representative immunohistochemistry stained images of (**j**-i) Hematoxylin-eosin, (**j**-ii) Moesin, (**j**-iii) Slug expression in A4 xenograft sections; black square indicates cells with giant nucleus. Scale bar- 40 μ.*p < 0.05, **p < 0.01.

WGCNA also established an association between module M5 expression and aneuploidy (Fig. 1c). Further detailed literature annotation assigned several functions including migration, invasion and metastases through EMT, change in morphology through cytoskeletal remodeling, enzymatic penetration of basal lamina, cell cycle, immune evasion to overcome host resistances and re-acquisition of ‘stemness’ features to ensure long-term cell survival and regeneration (Supplementary Table 4). Gene-gene interaction network analyses identified cytoskeletal remodeling genes as a major hub with several interacting partners (Supplementary Fig. 3a–c). Validation of core hub genes affirmed the association with aneuploid over euploid cells (Fig. 4a).

Towards a deeper study, we attempted establishment of single cell sorted aneuploid clones from xenografts. Interestingly, *in vitro* culture led to rapid genome stabilizion within 5–6 passages in cultured aneuploid cells, wherein all clones evolved to a near-euploid state indicating an inability to sustain the replicative stress imposed by aneuploidy (Supplementary Fig. 4a,b-i,b-ii). These aneuploid clones exhibited improved viability but not tumorigenic potential as compared to parental A4 cells, unless challenged with paclitaxel exposure (Supplementary Fig. 4c,d). These results suggest a role for dynamic niche signaling in maintenance of genetic instability besides confirming our earlier observations that the aneuploid fraction in naïve, untreated tumors is growth-arrested and exposure to stress and selective pressure triggers cell cycling and possibly selection^15^. In a separate study, we have also also shown that such aneuploid cells have potential to generate parallel drug resistant hierarchies within the same tumor^16^, that also emphasized microenvironmental stress to be essential for examining predicted functional correlates of aneuploid cells. We thus examined and compared the functional dynamics of aneuploid cells from naïve tumors with those under three- or six- doses of paclitaxel. Drug exposure not only enhanced frequency of aneuploidy (Supplementary Fig. 5a), but significantly contributed to enhanced *in vitro* regenerative potential as assayed by colony and spheroid forming assays (Fig. 4b–d; Supplementary Fig. 5b). Profiling of M5 genes in paclitaxel treated tumors also identified their upregulation in a dose-dependent manner (Fig. 4e) suggesting contributions to tumor cell survival. Most striking, expression of moesin and flotillin (known to contribute to centrosomal abnormilities, cytoskeletal remodelling and migration) were further enriched.

This led us to hypothesize that aneuploid cells in naïve tumors are ‘poised’ for metastases, which could be triggered by stress. On testing this conjecture through functional wound healing (migration) and matrigel invasion assays, aneuploid cells from paclitaxel treated xenografts indeed validated this predicted function (Fig. 4e–g; Supplementary Fig. 5c,d). In further quantifying cells with these enhanced capabilities, it was observed that the frequency of moesin, flotillin, and F-actin-moesin co-expressing cells (most likely to undergo metastases) were –

- (i) distinctly higher in aneuploid than euploid fractions in naïve tumors, and
- (ii) showed further enhanced expression following exposure to six doses of paclitaxel and
- (iii) represented the entire aneuploid population in treated tumors (Fig. 4h).

Concurrently acquisition of cellular plasticity is also evident with increased frequency of cells co-expressing fibroblast associated protein (FAP) and E-cadherin, indicating a propensity towards migration which is supported by the functional assays. A final visual evidence for cytoskeletal remodeling was provided by immunofluorescence profiling of F-actin and moesin expression in sorted euploid and aneuploid tumor cells subjected to scratch/wound healing assays (Fig. 4i). Presence and extensive re-arrangement of actin stress fibers and increased moesin expression was observed in aneuploid over euploid cells and provided visual evidence of active actin cytoskeletal remodeling.

Further, immunohistochemistry staining and analysis of A4 xenograft paraffin sections for moesin and Slug expression (a transcription factor involved in epithelial to mesenchymal transition – EMT; Fig. 4j-i,j-ii,j-iii) was also carried out along with detection of giant nuclei harboring cells that are likely to be aneuploid; expression quantification was carried out across 10 random fields at the migratory edge as well as at the core of tumors. This identified Slug and Moesin expression in aneuploid cells that were also more frequent at the migratory edge than in the tumor core (Supplementary Fig. 5e). Together, this affirms a latent metastatic potential in aneuploid cells, that may be activated by stress conditions through dynamic cell plasticity and modulation of the actin cytoskeleton.

## Discussion

From a wider perspective, our study provides some reasons for failure of biomarker and molecular target identification studies based on entire tumor profiling, which are also relevant to achieving complete elimination of tumor cells^29^,^30^. Recent approaches involving molecular profiling of mesenchymal/stromal cells, immune cells including macrophages and lymphocytes^31^,^32^,^33^ identify effects of inter-tumor heterogeneity by examining differences in tumors between different individuals. Cell sorting and differential fraction profiling has also been attempted in the context of CSCs *vs.* non-CSCs in tumors that unfortunately overlooks the role of progenitors and genetic instability in long-term cell survival and regeneration^34^,^35^. Closing the loop back to analyses of multiple-fraction sorted cells, we demonstrate how one can overcome limitations imposed by averaged measures to perform “reverse engineering” with molecular profiles of different cell types and identify their relevance to tumor behavior.

No direct correlation of CD53 expression with CSC activity is reported, yet our network analyses identified CD53 as a major hub in sorted CSC fractions. Apart from its conventional role in infiltration, modulating adhesion and migration of neoplastic B cells^36^, CD53 is also known to be associated with glioblastoma, ovarian, breast, colon, kidney, lung, uterine and rectum cancer^31^ wherein its expression correlates with radioresistance, matrix degradation, inflammation, regulating apoptosis to trigger tumor cell survival^37^,^38^ and results in poor patient prognosis^39^,^40^. Our study is possibly the first to identify and validate a strong correlation of CD53 with tumor regenerative capabilities. It also became apparent that cues for long-term regeneration and metastases are well established within the primary tumor as observed from activation of the actin cytoskeleton in quiescent, non-proliferative aneuploid cells by therapeutic stress. The latter also suggests that decoupling of moesin-flotillin-cytoskeletal remodeling from cell division may provide some advantages to migratory cells^41^.

Taken together and from a disease perspective, tumor initiating potential of CD53 expressing CSCs and progenitors could define tumor dormancy and recurrent disease, while aneuploid cells are likely to contribute not only through enhanced adaptation capabilities potentiated by their genetic diversity, but harbor a capability to escape from a restrictive or hostile microenvironment. Concurrently, with gain of regenerative potential this can lead to drug resistance, tumor metastases and disease relapse in ovarian cancer.

## Additional Information

**How to cite this article**: Naik, R. R. *et al.* Elucidation of molecular and functional heterogeneity through differential expression network analyses of discrete tumor subsets. *Sci. Rep.* **6**, 25261; doi: 10.1038/srep25261 (2016).

## Supplementary Material

Supplementary Information

## References

[b1] Frick P. L., Paudel B. B., Tyson D. R. & Quaranta V. Quantifying heterogeneity and dynamics of clonal fitness in response to perturbation. J Cell Physiol. 230, 1403–12 (2015). 25600161 10.1002/jcp.24888 PMC5580929

[b2] Tabassum D. P. & Polyak K. Tumorigenesis: it takes a village. Nat Rev Cancer. 15, 473–83 (2015). 26156638 10.1038/nrc3971

[b3] Singh A. K. *et al.* Tumor heterogeneity and cancer stem cell paradigm: updates in concept, controversies and clinical relevance. Int J Cancer. 136, 1991–2000 (2015). 24615680 10.1002/ijc.28804

[b4] Ajani J. A., Song S., Hochster H. S. & Steinberg I. B. Cancer stem cells: the promise and the potential. *Semin Oncol.* (2015) [Epub ahead of print]. Suppl 1:S3–17, doi: 10.1053/j.seminoncol.2015.01.001. 25839664

[b5] Fornara O. *et al.* Cytomegalovirus infection induces a stem cell phenotype in human primary glioblastoma cells: prognostic significance and biological impact. *Cell Death Differ.* (2015) [Epub ahead of print], doi: 10.1038/cdd.2015.91. PMC4716305 26138445

[b6] Preca B. T. *et al.* A self-enforcing CD44s/ZEB1 feedback loop maintains EMT and stemness properties in cancer cells. Int J Cancer. 137, 2566–77 (2015). 26077342 10.1002/ijc.29642

[b7] Zeijlemaker W. *et al.* A simple one-tube assay for immunophenotypical quantification of leukemic stem cells in acute myeloid leukemia. *Leukemia.* (2015) [Epub ahead of print], doi: 10.1038/leu.2015.252. 26437777

[b8] Zhang S. *et al.* Identification and characterization of ovarian cancer-initiating cells from primary human tumors. Cancer Res. 68, 4311–20 (2008). 18519691 10.1158/0008-5472.CAN-08-0364 PMC2553722

[b9] López J., Valdez-Morales F. J., Benítez-Bribiesca L., Cerbón M. & Carrancá A. G. Normal and cancer stem cells of the human female reproductive system. Reprod Biol Endocrinol. 19, 11, 53 (2013). 23782518 10.1186/1477-7827-11-53 PMC3693871

[b10] He Q. Z. *et al.* Isolation and characterization of cancer stem cells from high-grade serous ovarian carcinomas. Cell Physiol Biochem. 33, 173–84 (2014). 24504111 10.1159/000356660

[b11] Kakar S. S. *et al.* Withaferin a alone and in combination with cisplatin suppresses growth and metastasis of ovarian cancer by targeting putative cancer stem cells. Plos One 9, e107596 (2014). 25264898 10.1371/journal.pone.0107596 PMC4180068

[b12] Burgos-Ojeda D. *et al.* CD24+ Ovarian Cancer Cells Are Enriched for Cancer-Initiating Cells and Dependent on JAK2 Signaling for Growth and Metastasis. Mol Cancer Ther. 14, 1717–27 (2015). 25969154 10.1158/1535-7163.MCT-14-0607 PMC4496272

[b13] Martínez-Serrano M. J. *et al.* Is sphere assay useful for the identification of cancer initiating cells of the ovary? Int J Gynecol Cancer. 25, 12–7 (2015). 25365589 10.1097/IGC.0000000000000320

[b14] Zeimet A. G. *et al.* Ovarian cancer stem cells. Neoplasma. 59, 747–55 (2012). 22862176 10.4149/neo_2012_094

[b15] Kusumbe A. P. & Bapat S. A. Cancer stem cells and aneuploid populations within developing tumors are the major determinants of tumor dormancy. Cancer Res. 69, 9245–9253 (2009). 19951996 10.1158/0008-5472.CAN-09-2802

[b16] Naik R. R., Singh A. K., Mali A. M., Khirade M. F. & Bapat S. A. A tumor deconstruction platform identifies definitive end points in the evaluation of drug responses. *Oncogene.* (2015) [Epub ahead of print], doi: 10.1038/onc.2015.130. 25915841

[b17] Gerlinger M. *et al.* Intratumour heterogeneity in urologic cancers: from molecular evidence to clinical implications. Eur Urol. 67, 729–37 (2015). 24836153 10.1016/j.eururo.2014.04.014

[b18] Marusyk A. & Polyak K. Tumor heterogeneity: causes and consequences. Biochim Biophys Acta 1805, 105–117 (2010). 19931353 10.1016/j.bbcan.2009.11.002 PMC2814927

[b19] Yeh A. C. & Ramaswamy S. Mechanisms of Cancer Cell Dormancy-Another Hallmark of Cancer? *Cancer Res.* (2015) [Epub ahead of print], doi: 10.1158/0008-5472.CAN-15-1370. PMC4668214 26354021

[b20] Krem M. M., Press O. W., Horwitz M. S. & Tidwell T. Mechanisms and clinical applications of chromosomal instability in lymphoid malignancy. Br J Haematol. 171, 13–28 (2015). 26018193 10.1111/bjh.13507

[b21] Ben-David U. Genomic instability, driver genes and cell selection: Projections from cancer to stem cells. Biochim Biophys Acta. 1849, 427–35 (2015). 25132386 10.1016/j.bbagrm.2014.08.005

[b22] Bapat S. A., Mali A. M., Koppikar C. B. & Kurrey N. K. Stem and progenitor-like cells contribute to the aggressive behavior of human epithelial ovarian cancer. Cancer Res. 65, 3025–3029 (2005). 15833827 10.1158/0008-5472.CAN-04-3931

[b23] Zhao Y. & Simon R. BRB array tools data archive for human cancer gene expression: a unique and efficient data sharing resource. Cancer Informatics 6, 9–15 (2008). 19259398 10.4137/cin.s448 PMC2623314

[b24] Gardi N. L., Deshpande T. U., Kamble S. C., Budhe S. R. & Bapat S. A. Discrete molecular classes of ovarian cancer suggestive of unique mechanisms of transformation and metastases. Clin Cancer Res. 20, 87–99 (2014). 24132919 10.1158/1078-0432.CCR-13-2063

[b25] Langfelder P. & Horvath S. WGCNA: an R package for weighted correlation network analysis. BMC Bioinformatics 9, 559 (2008). 19114008 10.1186/1471-2105-9-559 PMC2631488

[b26] Margolin A. A. *et al.* ARACNE: an algorithm for the reconstruction of gene regulatory networks in a mammalian cellular context. *BMC Bioinformatics* **7**, (2006). (Suppl 1):S7, doi: 10.1186/1471-2105-7-S1-S7. PMC1810318 16723010

[b27] Huang da. W., Sherman B. T. & Lempicki R. A. Systematic and integrative analysis of large gene lists using DAVID bioinformatics resources. Nature Protocols 4, 44–57 (2009). 19131956 10.1038/nprot.2008.211

[b28] Hu Y. & Smyth G. K. ELDA: Extreme limiting dilution analysis for comparing depleted and enriched populations in stem cell and other assays. Journal of Immunological Methods 347, 70–78 (2009). 19567251 10.1016/j.jim.2009.06.008

[b29] Brown P. O. & Palmer C. The preclinical natural history of serous ovarian cancer: defining the target for early detection. Plos Medicine 6, e1000114 (2009). 19636370 10.1371/journal.pmed.1000114 PMC2711307

[b30] Bapat S. A., Krishnan A., Ghanate A. D., Kusumbe A. P. & Kalra R. S. Gene expression: protein interaction systems network modeling identifies transformation-associated molecules and pathways in ovarian cancer. Cancer Res 70, 4809–19 (2010). 20530682 10.1158/0008-5472.CAN-10-0447

[b31] Andreopoulos B. & Anastassiou D. Integrated Analysis Reveals hsa-miR-142 as a Representative of a Lymphocyte-Specific Gene Expression and Methylation Signature. Cancer Inform. 11, 61–75 (2012). 22570537 10.4137/CIN.S9037 PMC3306237

[b32] Verardo R. *et al.* Specific mesothelial signature marks the heterogeneity of mesenchymal stem cells from high-grade serous ovarian cancer. Stem Cells. 32, 2998–3011 (2014). 25069783 10.1002/stem.1791

[b33] Busuttil R. A. *et al.* A signature predicting poor prognosis in gastric and ovarian cancer represents a coordinated macrophage and stromal response. Clin Cancer Res. 20, 2761–72 (2014). 24658156 10.1158/1078-0432.CCR-13-3049

[b34] Schwede M. *et al.* Stem cell-like gene expression in ovarian cancer predicts type II subtype and prognosis. Plos One 8, e57799 (2013). 23536770 10.1371/journal.pone.0057799 PMC3594231

[b35] Vathipadiekal V. *et al.* Identification of a Potential Ovarian Cancer Stem Cell Gene Expression Profile from Advanced Stage Papillary Serous Ovarian Cancer. Plos One 7, e29079 (2012). 22272227 10.1371/journal.pone.0029079 PMC3260150

[b36] Barrena S. *et al.* Aberrant expression of tetraspanin molecules in B-cell chronic lymphoproliferative disorders and its correlation with normal B-cell maturation. Leukemia 19, 1376–83 (2005). 15931266 10.1038/sj.leu.2403822

[b37] Finis K. *et al.* Analysis of pigmented villonodular synovitis with genome-wide complementary DNA microarray and tissue array technology reveals insight into potential novel therapeutic approaches. Arthritis Rheum. 54, 1009–19 (2006). 16508983 10.1002/art.21641

[b38] Yunta M. & Lazo P. A. Apoptosis protection and survival signal by the CD53 tetraspanin antigen. Oncogene 22, 1219–24 (2003). 12606948 10.1038/sj.onc.1206183

[b39] Domingues P. H. *et al.* The protein expression profile of meningioma cells is associated with distinct cytogenetic tumour subgroups. Neuropathol Appl Neurobiol. 41, 319–32 (2015). 24612434 10.1111/nan.12127

[b40] Khirade M. F., Lal G. & Bapat S. A. Derivation of a fifteen gene prognostic panel for six cancers. *Sci Rep.* (2015). [Epub ahead of print], doi: 10.1038/srep13248. PMC4536526 26272668

[b41] Larson S. M. *et al.* Cortical mechanics and meiosis II completion in mammalian oocytes are mediated by myosin-II and Ezrin-Radixin-Moesin (ERM) proteins. Mol Biol Cell. 21, 3182–92 (2010). 20660156 10.1091/mbc.E10-01-0066 PMC2938384

