# Supplementary material for: Elucidation of molecular and functional heterogeneity through differential expression network analyses of discrete tumor subsets
Source: Sci Rep. 2016 May 3;6:25261. doi: 10.1038/srep25261 (PMC4853737; doi:10.1038/srep25261)
Supplement: Supplementary Information [file srep25261-s1.pdf]

Supplementary Information

Title- Elucidation of molecular and functional heterogeneity through differential expression network analyses of discrete tumor subsets

Authors- Rutika R.Naik, Nilesh L. Gardi, Sharmila A. Bapat.

<u>**Supplementary Figures**</u>

Supplementary Fig.1

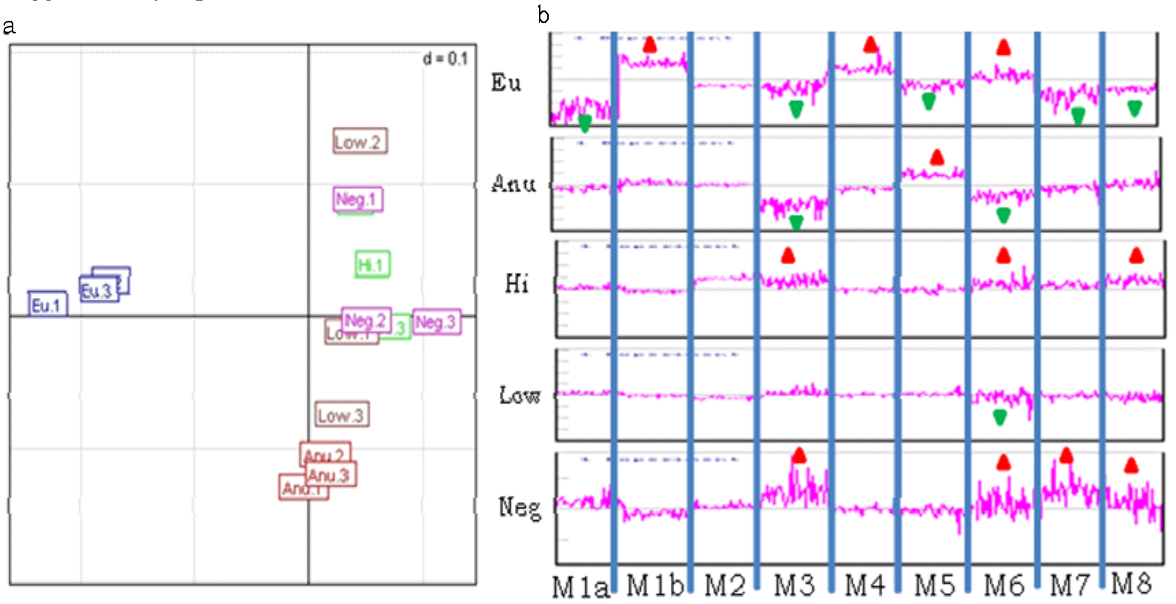

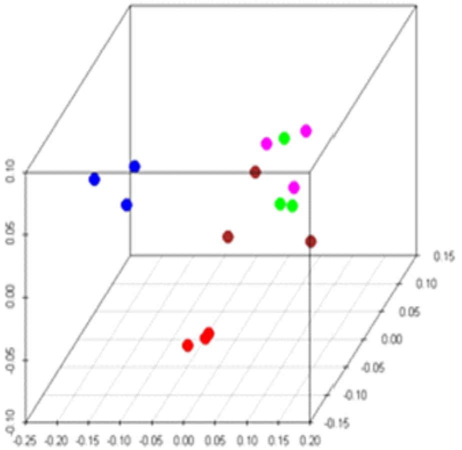

	↑	↓
PKH^{hi}	M2,M3,M6, M7M8	-
PKH^{lo}	-	M6
PKH^{neg}	M3,M7,M8	M6
Euploid	M1b,M4, M6	M1a,M3, M5.M7,M8
Aneuploid	M5	M3,M6

Supplementary Fig.1. (**a**) Two- (upper panel) and Three- (lower panel) dimensional plots of unsupervised Principle component analysis (PCA) exhibiting distinct clusters with projection of 5 samples (Euploid, Aneuploid, PKHhi, PKHlo, PKHneg) represented in blue , red, green, brown and pink colors respectively.Table : Enriched module genes specific to each class; (**b**) (upper panel) Representative WGCNA module enrichment in 5 tumor samples (Euploid, Aneuploid, PKHhi, PKHlo, PKHneg) ; red and green triangles indicate upregulated and downregulated modules respectively, (**b**) (lower panel) Distribution of WGCNA modules based on expression across tumor subsets where ↑ and ↓ represents up-regulation and down regulation respectively.

Supplementary Fig.2

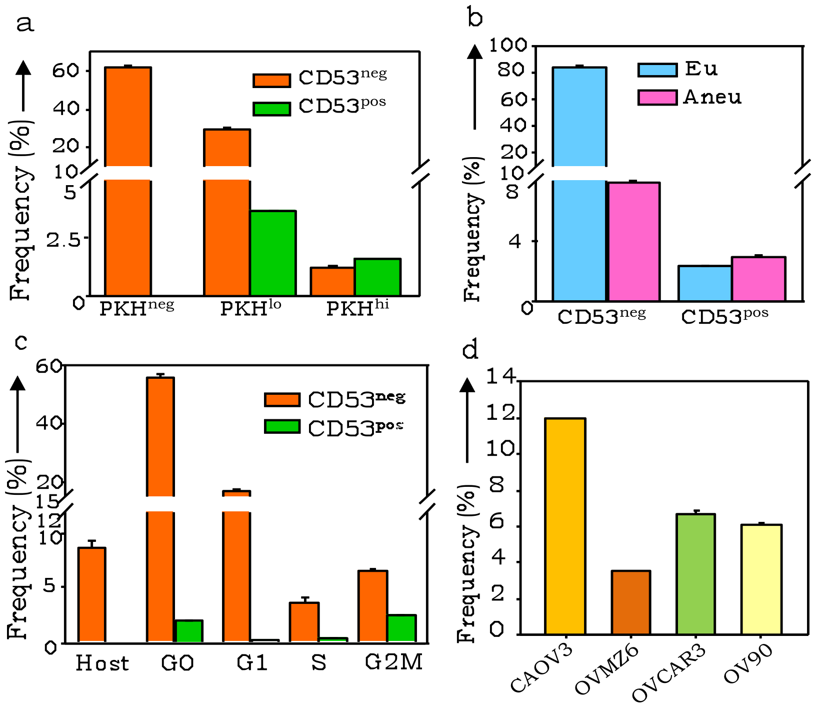

Supplementary Fig.2. Frequency of expression of CD53 in (**a**) PKH derived CSC hierarchy (PKHhi PKHlo PKHneg); (**b**) Genetic instability derived subsets; (**c**) cell cycle phases (G0,G1,S,G2M) and host cells in A4 xenograft; (**d**) Graphical representation of expression of CD53 in xenografts generated from ovarian cancer cell lines CAOV3, OVMZ6, OVCAR3 and OV90.

Supplementary Fig.3

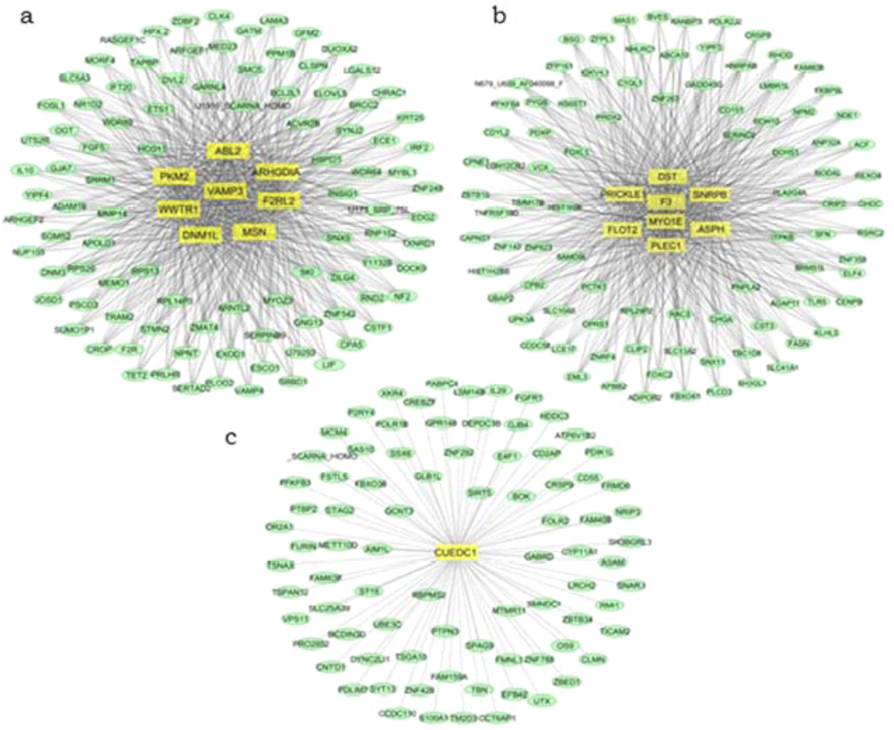

Supplementary Fig.3. Gene – gene interaction AracNe network analysis of M5 cytoskeletal remodeling genes with (**a**), ABL2, F2RL2, MSN, WWTR1, PKM2, VAMP3, ARHGDIA, DNML1; (**b**), DST, F3, FLOT2, ASPN, PLEC1, SNRPB, PRICKLE, MYO1E; (**c**) CUEDC1 as nodes.

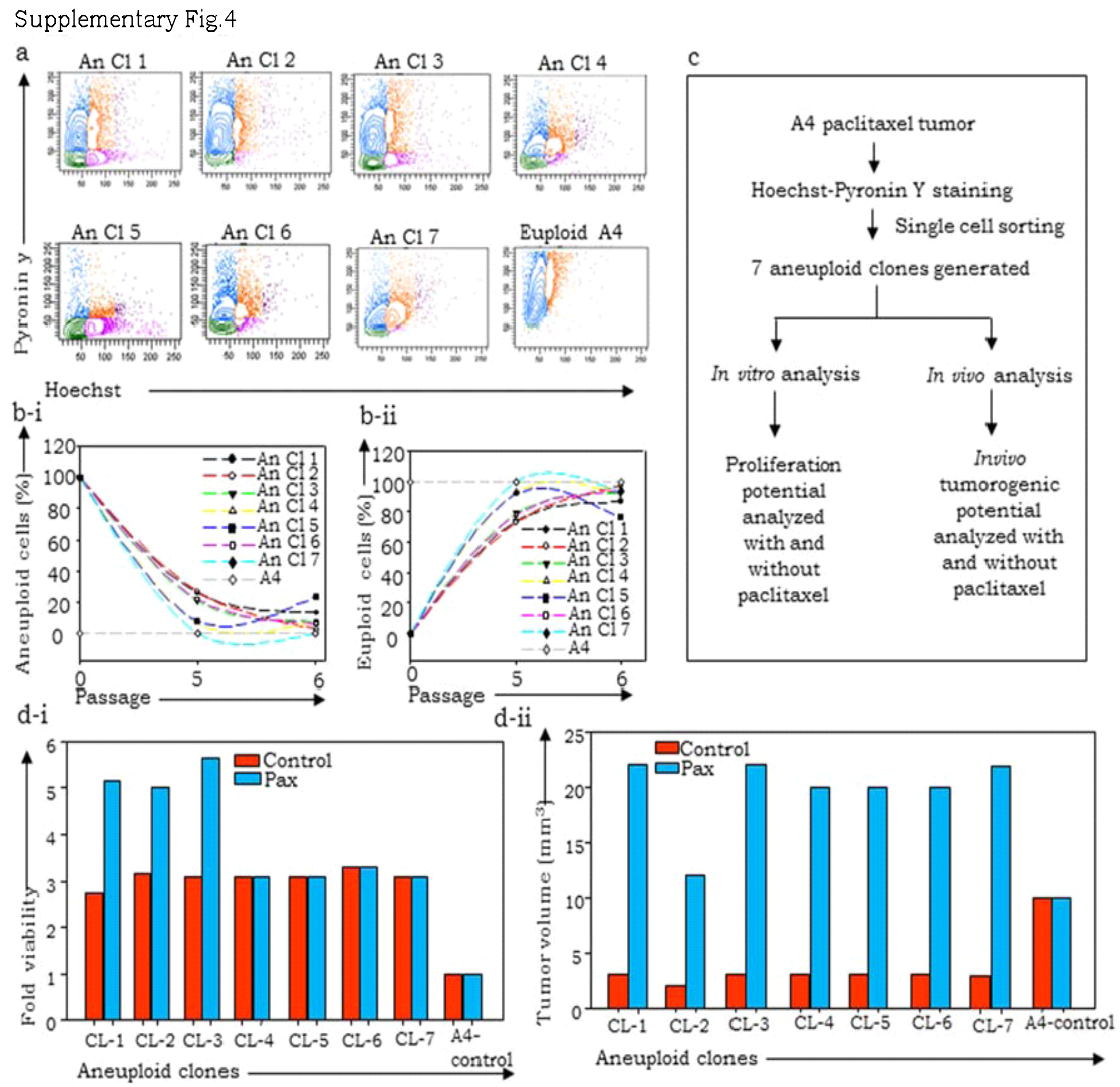

Supplementary Fig.4. Behavior of aneuploid cells (**a**) Representative FACs profile of Hoechst Pyronin Y staining of single cell sorted aneuploid clones at passage 6, A4 cell line used as euploid control; (**b**) Frequency of aneuploid and euploid cells in each clone at passages 5 & 6; (**c**) Outline of *in vitro* and *in vivo* analysis of aneuploid clones; (**d-i**) Cell viability of 7 aneuploid clones and A4 control cells evaluated through trypan blue staining, fold-change calculated with respect to A4 cells; (**d-ii**) *In vivo* tumorigenecity assay by injecting 7 aneuploid clones and A4 control cells s.c in NOD/SCID mice with and without paclitaxel exposure.

Supplementary Fig.5

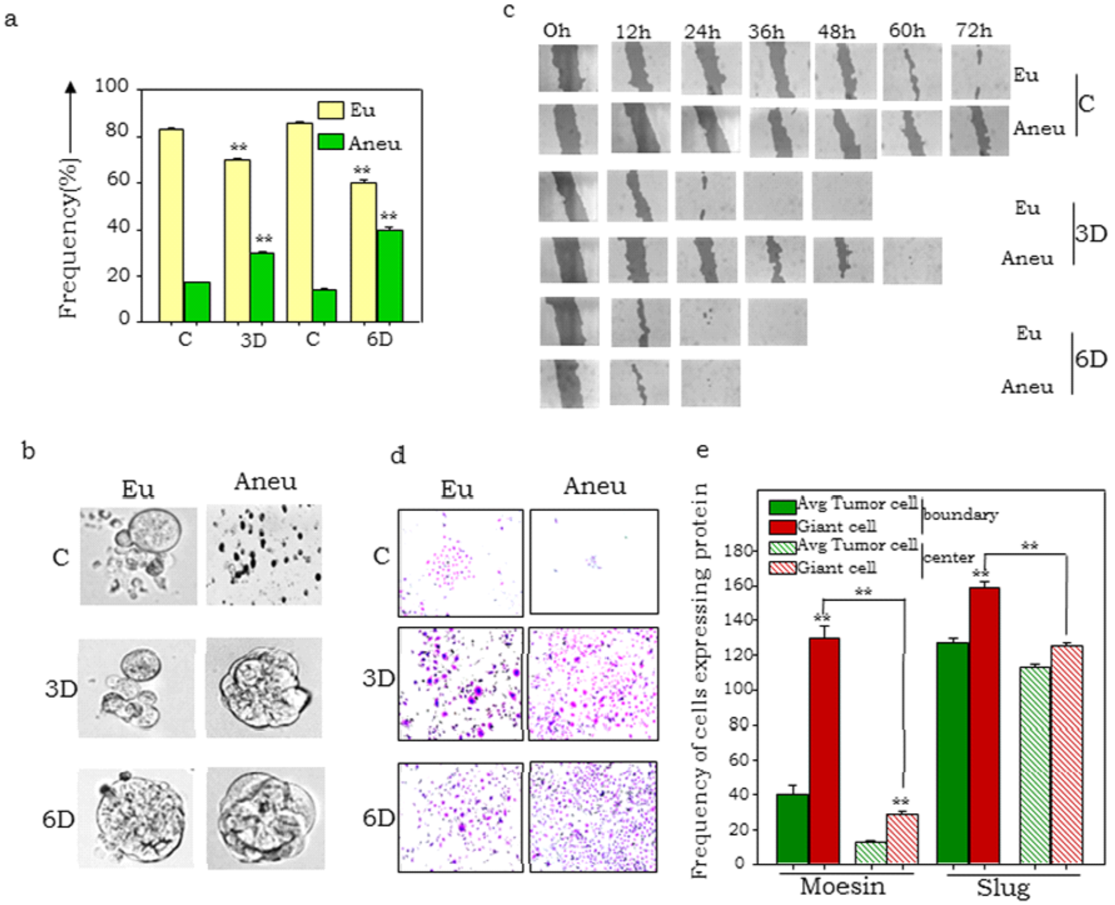

Supplementary Fig.5(**a**) Frequency of euploid and aneuploid cells in tumors indicating extent of genetic instability in naïve and paclitaxel treated A4 xenografts. (**b**) Representative images of spheroids from sorted euploid and aneuploid cells; (**c**) Representative images of scratch assay using T-scratch software where C- Control, 3D- Paclitaxel 3D and 6D- Paclitaxel 6D treated xenografts; (**d**) Crystal violet stained image of migratory cells (matrigel invasion assay); (**e**) Frequency of slug and moesin expression in cells varying in their DNA content identified through giant and average size nuclei in immunohistochemistry stained sections, 10 random fields each at the migratory edge and within tumor core and boundary respectively were scored using ImageJ; *p<0.05, **p<0.01.

Supplementary Tables

Supplementary Table 1. Module genes

M1a	M1b	M2	M3	M4	M5	M6	M7	M8
ZNF66	LMCD1	SLC6A14	CASP14	PDCL	PSCD3	HILS1	FAM138E	CD3D
ZNF770	TXNDC5	AAA1	SHANK1	MRPL1	SERPINB9	AIF1	DRD2	CDKN2BAS
ZNF708	JAG1	FFAR3	GGTL4	ACTR6	ZBTB10	PVALB	HAMP	CCDC13
ZNF141	SQRDL	ASPA	NPBWR2	UBP1	UBE3C	NR2E1	TGM4	SLITRK2
ZNF254	CECR5	SERPINA13	OR52K2	SNX6	RPL14P3	BSND	GRM2	ASCL4
ZNF486	SLC25A15	RHD	PRO0478	SIP1	PLEC1	PANK1	FBXO40	PRKCQ
ZNF468	SLC19A2	CD53	MCCD1	TMCO1	BRCC2	PARD3	LILRP2	DDX3Y
ZNF714	ZNF268	IGHV6.1	HAAO	CAMSAP1L1	DST	JMJD2C	LILRA1	KLRB1
FBXO11	DYNLT3	CCL18	STRA6	KRCC1	FGF5	SLC12A3	EVI2A	Z99394
ZNF417	MRPS9	GRM6	RP5.1054A22.3	TGDS	ABL2	AGRP	PSG7	NCAM1
LTBP4	LAS1L	TAS2R38	HBM	HIBCH	VAMP3	TRPC7	HCG8	SLC6A11
ZNF681	SMYD2	KRT8P25	GML	NFYB	DNM1L	CD8B	PACRG	NLRP13
IFRG15	FRG1	SLC35D3	TSPAN32	NMI	PKM2	ADORA1	GPR119	U640_FMR1P
ZBTB37	LSM1	CYP4F22	CSNK1A1L	DPM1	CUEDC1	DPP6	SNX32	ESYT3
ZNF117	ECHDC1	SERPINA11	SPACA3	E2F3	MCM4	MPPED1	CYP17A1	GNRHR
MLL	IFNGR1	S100A7A	CYB561D1	ZNF830	ELOVL5	RP11.49G10.8	OTOS	TRIML1
PCDHGB4	NAP1L1	OR2W1	SLC1A7	PPIC	F3	BARHL2	MN1	OTOR
SMA4	CBX3	OR2A14	CCDC8	NSUN3	MSN	KCNA7	DCST2	SERPIND1
DDX6	SEC11A	HCRTR2	GPR17	CCDC6	SLC5A3	TNR	CDK5R2	CEACAM5
UHMK1	DNAJB6	KLRK1	PRM1	KLHL28	GJA7	LCE3A	BAI1	KCNU1
PCF11	ETHE1	CATR1	SH2D3C	PLSCR1	NR1D2	IGHV3.48	OR8B4	HOXA9
ZNF429	USP1	TTTY12	ZSCAN1	WASL	ARHGDIA	DKK4	C1QC	SCUBE1
POTE15	TXNL2	OR10Q1	CYP2W1	ANXA7	ASAM	RFPL1	DEFA1B	SLCO6A1
ZNF85	HECA	MMP26	KLRC4	ZHX1	ASPH	TNFSF11	ZNF157	MSMB
ZNF813	SCHIP1	P2RY14	FES	TRIM13	CDYL2	NLF2	SNCB	PCDHGA5
POTE2	POLR3GL	APOC3	SLC6A19	TFAM	PNPLA2	SPACA5	MYCN	PKD1L3
ZNF772	PCTP	KRT1	PRRG3	SATB1	HIST1H2BB	C1QL2	CACNG4	GPR115
THRAP1	UCK2	GBP6	MPFL	KTN1	U1916_SCARNA	SUNC1	TMEM63B	OR52E6
GNS	CLNS1A	GNAT2	LCE6A	MSH6	SNRPB	GYPE	H1FNT	ANKRD1
NCK2	PAIP2	TCEB3B	R78313	IFI16	FLOT2	SELV	STEAP4	IFNA14
ZNF283	RAD21	CD84	ANTXRL	CCDC41	ZNF140	PDZD3	N776_U733_ AF079515_S	GALP
ZNF257	ITGA6	DNAJB7	TRBV5.4	PRPF39	ZNF542	DCDC2B	MSI1	PAGE1
ZNF569	LEPRE1	KLHL32	IYD	TRUB1	RDH10	CSPG4LYP1	WDR87	CHRNG
ZNF675	SEH1L	U1058_PCGEM1	CRHR1	PPP3CA	OPRS1	TNP1	SLC22A18AS	KLK7
UBE1L2	MED4	GRM1	DEF6	ECD	CRSP8	LEPR	COL10A1	MSMP
ZNF676	PPP2CB	OR10R2	MYADML	ZNF654	KLHL5	PGBD3	ROGDI	ADAM30
EIF2S3	HYPK	BTLA	CTSG	TRIT1	WWTR1	PIWIL2	VGF	U82313
DPEP3	CDK7	OR8K3	C4BPA	RAP2A	PRICKLE1	KCNQ1DN	ANK2	MYPN
THAP5	PECI	TAS1R2	ENPP4	MCCC1	SLC10A5	SLC30A10	GORASP1	GJA9
ZNF460	FBXO21	TRAT1	CREB3L3	ARMC8	TXNRD1	VENTXP7	VPREB3	
ACTBL1	PWP1	SEZ6L	MC5R	SNAPC1	ZNF788	PRSS7	SLC13A1	
MYO1B	LARP2	OR7A5	NMUR1	NOC3L	MYO1E	SMPX	U09197	
SLC2A3P1	SNX14	MYOM2	OR6C3	NCBP1	F2RL2	PLA2G12B	OR2T3	
LGR5	RABGGTB	STK32B	PAX7	FANCL	HSPD1	TBX3	IL1F6	
ZNF652	RRM1	SLC17A6	PRDM7	RUNDC1	HIST1H3I	PNMA3	FRMPD3	
BCL9	PNRC2	PTPN20B	CSN1S1	CFDP1	SGMS2	CLEC4G	NUMBL	
ZNF595	NUF2	CLEC3A	HCK	MAT2B	EDG2	UBL4B	DAPP1	
PDZD8	MAD2L1BP	ABCB11	SLC2A4	ZCWPW1	RPS13	KDR	FUT7	
ZNF146	OCIAD1	BTG4	KCNN2	ZFAND6	RBPMS2	SYT2	TMEM174	
RALGPS2	SNRPB2	CXCL6	IGHA2	LYST	ETS1	GRHL1	OR51B4	

Supplementary Table 2. DAVID pathway analysis of M2 and M5 genes

Pathway	Genes
**M2 – Cluster 1 (Cl1)**	
G-protein coupled receptor signaling	ABCA1, PDYN, GPR101, GRP, APOC3, CCR2, CGB, CRH, GNAO1, MS4A2, OR1E1, OR1N1, OR10AG1, OR10G3, OR10R2, OR11G2, OR4C13, OR4C6, OR4D9, OR4K14, OR4K15, OR4S2, OR5AP2, OR5H15, OR51I1, OR51T1, OR51T1, OR52A1, OR52A5, OR52A5, OR52K1, OR7A5, OR8A1, OR8I2, OR8K3, OR9Q1, OR9Q1, RGS18, VN1R2, VN1R4, TAS1R2, TAS2R38, TAS2R41
Integral to membrane signaling	ABCA1, ATP3A4, BTLA, BOC, CLEC17A, CD1C, CD300LG, CD53, AAA1, EPHA3, EPHA8, FAT3, FLT1, FCGR1B, GPR101, GRAMD2, KCNS1, MPV17L,GALNT13, ATP13A4, B3GALT2, GRIN2A, RHD, GALNTL6, WSCD1, ACSL6, AJAP1, AMOT, ASPN, CDH9, CLSTN2, CEACAM7, CCR2, CATSPERG, CCDC60, COLEC12, DCC, EIF5AL1, FLVCR2,FMO3, GRIA2, GRIN2A, ENPEP,PRPH2, IGSF9B, ITGA8, IZUMO1, LRK1,LRRC15,LRRC52, MS4A2, MS4A3, MS4A8, BNLGN4X, KCNB2, OR1E1, OR1N1, OR10AG1, OR10G3, OR10R2, OR11G2, OR4C13,OR4C6,OR4D9, OR4K14, OR4K15, OR4S2, OR5AP2, OR5H15, OR51I1, OR51T1, OR52A1, OR52A5, OR52K1, OR7A5, OR8A1, OR8I2,OR8K3,OR9Q1PRPH2, KCNB2, KCNS1, KCNB2, KCNH7, PDCD1LG2, PPP1R3A, PTPRO, RET, RNF148, RNF222, SIGLEC, VN1R2, VN1R4, SLC22A3, SLC25A18, SLC26A5, SLC26A9, SLC28A3, SLC6A2 ,SLCO1C1, TAS1R2, TAS2R38, TAS2R41, TLR7, TCEB3B, TMEM204, AJAP1, TNFRSF1B, ERG, VN1R2, VN1R
Meiotic cell cycle	BOLL,FMN2,GRP,SPDYA ,SLC26A8, TEX11
**M2 – Cluster 2 (Cl2)**	
Transmembrane	ABCA8,CD96,EPHB6,GPR1,TSC22D3,BDKRB1,EMCN,GAL3ST3,KIR3DP1,KLRF1, LTC4S,OR1S1,OR13F1,OR5AS1,KCNMB2
Integral to membrane	CD96,KLRF1,LTC4S, LTC4S, OR1S1, OR13F1, OR4D11, OR5AS1, OR8D4, KCNMB2, ABCA8
G-protein coupled receptor protein signaling pathway	GPR1, BDKRB1, OR1S1, OR13F1, OR4D11, OR5AS1, OR8D4
Transducer signaling pathway	BDKRB1, OR1S1, OR13F1, OR4D11, OR5AS1, OR8D4, GPR1
**M2 – Cluster 3 (Cl3)**	
Transducer signaling	GPR123, GPR27, GPR97, RHO, MRGPRX1, MRGPRX4, ADRA2B,CNR1,CCR3, CCKBR, DRD1, FSHR, FFAR3, FZD10, GABBR2, GRM1, GRM3, GRM6, GNAT2, HCRTR2, P2RY12, OPRD1, GRM3, OR1B1, OR1L4, OR10A6, OR10A7, OR10Q1, OR10T2, OR10W1, OR11H6, OR12D3, OR13C4, OR13D1, OR13J1, OR2A12, OR2A14, OR2AK2, OR2G3, OR2G6, OR2M4, OR2T6, OR2W1, OR4B1, OR4C3, OR4D10, OR4K5, OR4M2, OR4N5, OR5AK3P, OR5AU1, OR5B21, OR5D18, OR5J2, OR5M1, OR5M10, OR5W2, OR51D1, OR52H1, OR52J3, OR52N4, OR52W1, OR56A1, OR6A2, OR6C75, OR6K6, OR8B3, OR8G5, OR8H2, OR9A2, P2RY14, RGS7, SPATA22
G-Protein coupled receptor protein signaling pathway	GPR123, GPR27,GPR97, RHO, MRGPRX1, MRGPRX4, ADRA2B, CNR1, CCL3, CCR3, CXCL6, CCKBR, DRD1, EMR4P, FSHR, FFAR3, FZD10, GABRA1, GABBR2, GRID2IP, GRM1, GRM3, GRM6, GNAT2, HCRTR2, P2RY12, OPRD1, CCR3, GRM3, OR1B1, OR1L4, OR10A6, OR10A7, OR10Q1, OR10T2, OR10W1, OR11H6, OR12D3, OR13C4, OR13D1, OR13J1, OR2A12, OR2A14, OR2AK2, OR2G6, OR2M4, OR2T6, OR2W1, OR4B1, OR4C3, OR4C45, OR4D10, OR4K5, OR4M2, OR4N5, OR5AK3P, OR5AU1, OR5B21, OR5D18, OR5J2, OR5M1, OR5M10, OR5W2, OR51D1, OR5M1, OR5M10, OR52H1, OR52J3, OR52N4, OR52N5, OR52W1, OR56A1, OR6A2, OR6C75, OR6K6, OR8B3, OR8G5, OR8H2, OR9A2, OPRD1, PYY, PDC, P2RY12, P2RY14, QRFP, RGS7, RGS4, RGS7, EMR4P, SPATA22, VIP, HTR3B, EMR4P

GPCR, rhodopsin-like	GPR27, RHO, MRGPRX1, MRGPRX4, ADRA2B, CNR1, CCR3, CCKBR, DRD1, FSHR, FFAR3, HCRTR2, P2RY12, OPRD1, OR1B1, OR1L4, OR10A6, OR10A7, OR10Q1, OR10T2, OR10W1, OR11H6, OR12D3, OR13C4, OR13D1, OR13J1, OR2A12, OR2A14, OR2AK2, OR2G3, OR2G6, OR2M4, OR2T6, OR2W1, OR4B1, OR4C3, OR4D10, OR4K5, OR4M2, OR4N5, OR5AK3P,OR5AU1, OR5B21, OR5D18, OR5J2, OR5M1, OR5M10, OR5W2, OR51D1, OR52H1, OR52J3,OR52N4,OR52W1, OR56A1, OR6A2, OR6C75, OR6K6, OR8B3, OR8G5, OR8H2, OR9A2, OPRD1, P2RY12, P2RY14, RHO, SPATA22
Intrinsic to membrane	GPR27, GPR97, HIGD2B, TRPC5, RHO, LCP2, HTR3B, ABO, ADAM7, CCR3, ABCB11, CLEC4D, CD5, CD79B,CD84, CD93, CD79B, EMR4P, FCGR3B, GPR123, GPR27, GPR97, TRPC5, RHO,T,MRGPRX1, MRGPRX4, MAG, MYADML2, NAALADL1, NAT8B, NOX3, NIPAL4, PMP22CD, SIDT1, SVOP, TRAT1, TARP, TMEM82, UGT3A1, GALNTL5, HVCN1, ADAM7, ADIG, ADRA2B, ACCN5, ARMCX1, ABCB11, BTNL3, CACNA1S, CNR1, CCR3, CLIC6, CCKBR, CLRN1, CSF3R,C8B, CNTNAP4, CNNM1, DRD1, EMR4P, TRPV5, EXTL1, FLRT2, FSHR, FFAR3, FZD10, GABRA1, GABBR2, MAGEA9B, GJE1, GRM1, GRM3, GRM6, GYPB, LILRB4, GUCY2D, HHATL, HFE2, HVCN1, HCRTR2, KCNJ10, P2RY12, TMEM82, OPRD1, TMEM163, CCR3, MUSK, SLC17, A6HHATL, IL1RL2, IMPG2, KIR2DL4, KLRC3, LRRC3, LILRB4, LRP1B, LAX1, LTA, MUC21, MUC4, MYADML2, NETO1, OR1B1, OR1L4, OR10A6, OR10A7, OR10Q1, OR10T2, OR10W1, OR11H6, OR12D3, OR13C4, OR13J1, OR2A12, OR2A14, OR2AK2, OR2G3, OR2G6, OR2M4, OR2T6, OR4B1, OR4C3, OR4C45, OR4D10, OR4M2, OR4N5, OR5AK3P, OR5AU1, OR5B21, OR5D18, OR5J2, OR5M1, OR5M10, OR5W2, OR51D1, OR52H1, OR52J3, OR52N4, OR52W1, OR56A1, OR6A2, OR6C75, OR6K6, OR8B3, OR8G5, OR9A2, OPRD1, PTCHD2, KCTD16, KCNT2, KCNJ5, KCNA6, KCNG2, KCNH1, CSF3R, PTPRT, P2RY12, P2RY14, RHO, SLFN12L, SEZ6L, LRRC3, EMR4P, KCTD16, SCN3B, SLC17A1, SLC17A6, SLC18A3, SLC24A5, SLC30A8, SLC35D3, SLC6A14, SLC6A18, SLC9A11, SPATA22, SUSD4, SVOP, SYT6, SYT6, TPO, TRPC5, TRPV5, TMC1, TMPRSS7, TMEM100, TMEM155, TMEM163, TMEM82, TYR, ZP4, HTR3B
Ion channel activity	HTR3B,TRPC5,HVCN1,ACCN5,CACNA1S,CLIC6,TRPV5, GABRA1, MAGEA9B, GJE1,HVCN1,KCNJ10,KCTD16,KCNT2,KCNJ5,KCNA6,KCNG2,CNH1,SCN3B, SLC9A11
Passive transmembrane transporter activity	HTR3B,TRPC5,HVCN1,ACCN5,CACNA1S,CLIC6,TRPV5,GABRA1,MAGEA9B, HVCN1, KCNJ10, KCTD16, KCNT2, KCNJ5, KCNA6, KCNG2, KCNH1, KCTD16, SCN3B, SLC6A18, SLC9A11, TRPC5, TRPV5
Gated channel activity	HTR3B,HVCN1, ACCN5, CACNA1S, CLIC6, GABRA1, MAGEA9B, HVCN1, KCNJ10, KCTD16, KCNT2, KCNJ5, KCNA6, KCNG2, KCNH1, KCTD16, SCN3B, TRPC5
Cation channel activity	HTR3B, ACCN5, CACNA1S,TRPV5,HVCN1, KCNJ10, KCTD16, KCNT2, KCNJ5, KCNA6, KCNG2, KCNH1,KCTD16,SCN3B, TRPC5, TRPV5
Voltage-gated ion channel activity	HVCN1, CACNA1S, CLIC6, KCNJ10, KCTD16, KCNJ5, KCNA6, KCNG2, KCNH1, KCTD1, SCN3B
Extracellular ligand-binding receptor	GABBR2, GRM1, GRM3, GRM6, GUCY2D
GPCR, family 3, metabotropic glutamate receptor	GRM1, GRM3, GRM6
**M5 – Cluster 1 (Cl1)**	
Ezrin / radixin / moesin (ERM)	PTPN3, MSN, NF2, PTPN3
**M5 – Cluster 2 (Cl2)**	
Cytoskeletal protein binding	CLIP2, ANP32A, DST, MYO1E, NDE1
**M5 – Cluster 3 (Cl3)**	
Cell projection assembly	CD2AP,DYNC2LI1,TSGA10

Supplementary Table 3. Literature annotation of biological functions associated with top 50 M2 genes

Cell survival	Cell adhesion and migration	Stem cell function, differentiation Cell cycle arrest	Immune modulation evasion and inflammation	Transformation and Tumor progression	Therapy resistance	Drug or therapy target	Solute trasporter	Olfactory receptor and Taste receptor signalling	DNA damage or DNA assembly	Epigenetic regulation	lncRNA
CD53	CD53	CD53	CD53	S100A7A	P2RY14	CYP4F22	SLC6A14	OR2W1	DNAJB7	GRM6	TTTY12
CD84	CCL18	P2RY14	CCL18	CATR1	ABCB11	SERPINA11	SLC35D3	OR2A14	AAA1	TCEB3B	KRT8P25
TRAT1	S100A7A	ASPA	S100A7A	GRM1	ASPA	SLC6A14	SLC17A6	OR10R2			U1058_PCGEM1
	MMP26	BTG4	BTLA	STK32B	KLRK1	HCRTR2		OR8K3			
	STK32B	KRT1	KLRK1	ASPA		APOC3		OR7A5			
	CLEC3A		CXCL6			ASPA		GNAT2			
	CXCL6		GBP6			GRM1		TAS2R38			
			STK32B			FFAR3		TAS1R2			
			GRM1								
			FFAR3								
			SEZ6L								
			P2RY14								
			MMP26								
			CD84								
			TRAT1								

M2 Genes with unknown functions - SERPINA13, RHD, IGHV6.1, MYOM2, PTPN20B, OR10Q1, KLHL32

Supplementary Table 4. Literature annotation of biological functions associated with top 50 M5 genes

Migration / metastases / cytoskeleton	cell cycle / senescence/ Aneuploidy	Immune modulation / Apoptosis evasion	Transcription factors	Stem cells and differentiation	Metabolism	Lipid and cholestrol metabolism	Chromatin remodeling	Ribosome assembly/ Proteosome assaciated
PLEC1	PSCD3	SERPINB9	ZBTB10	FGF5	PKM2	VAMP3	HIST1H2BB	RPL14P3
DST	VAMP3	DNM1L	DNM1L	DNM1L	SLC5A3	ELOVL5	HIST1H3I	RPS13
ABL2	DNM1L	OPRS1	OPRS1	CDYL2	NR1D2	PNPLA2		RBPMS2
VAMP3	BRCC2	TXNRD1	TXNRD1	WWTR1	SLC10A5	SGMS2		UBE3C
DNM1L	MCM4	HSPD1	HSPD1	ETS1	TXNRD1	EDG2		
PKM2	ARHGDIA							
CUEDC1	RDH10							
F3								
MSN								
ASPH								
FLOT2								
WWTR1								
MYO1E								
F2RL2								
SNRPB								
ARHGDIA								
PRICKLE1								

M5 Genes with unknown functions - GJA7, ASAM, U1916, SCARNA, CRSP8, KLHL5
